# Risk factors and predictive model construction for severe complications following repair of perforated peptic ulcer

**DOI:** 10.3389/fsurg.2025.1656837

**Published:** 2025-10-14

**Authors:** Qinfeng Sun, Yujie Kui, Federico Maria Mongardini, Ludovico Docimo, Qianqian Zhang, Xiaoxia Xu, Junfeng Li, Kailian Qiu, Zechao Zhu

**Affiliations:** 1 Haiyan People’s Hospital, Jiaxing, China; 2 University of Study of Campania "Luigi Vanvitelli", Naples, Italy; 3 The Second Affiliated Hospital of Jiaxing University, Jiaxing, China

**Keywords:** peptic ulcer, perforated peptic ulcer, postoperative complications, nomogram, risk factor

## Abstract

**Objective:**

To identify risk factors and construct a prediction model for severe complications(Clavien-Dindo Classification III-V)following repair of perforated peptic ulcers.

**Methods:**

Clinical data from 230 patients who underwent perforated peptic ulcer repair at Haiyan County People's Hospital and Jiaxing Second Hospital between January 2018 and June 2024 were retrospectively analyzed. Univariate and multivariate logistic regression analyses were performed to screen relevant variables, followed by the development of a risk prediction model for severe complications, with predictive performance validated using receiver operating characteristic (ROC) curve analysis.

**Results:**

The cohort comprised 230 patients (185 males, 45 females) with a mean age of 62.2 ± 17.8 years, predominantly presenting with gastric perforations. Severe postoperative complications occurred in 42 cases (18.3%). In ERAS patients, advantages were observed in terms of the incidence of severe complications and length of hospital stay; however, these differences did not reach statistical significance. Analytical results indicated that alcohol use history, ASA score, admission nutritional score, CRP level, and preoperative albumin level were independent risk factors for severe complications (*P* < 0.05). The nomogram constructed based on multivariate analysis showed excellent discriminative ability (AUC = 0.961), with calibration curves indicating good agreement between predicted and observed outcomes. Decision curve analysis confirmed the clinical utility of this model.

**Conclusion:**

This prediction model demonstrates high accuracy for severe complications after peptic ulcer perforation repair, providing valuable guidance for clinical monitoring and early preventive interventions.

## 1 Introduction

Peptic ulcer (PU) is a highly prevalent condition in daily life, with a lifetime prevalence of 5%−10% and an annual incidence of 0.1%−0.3% in the general population (1). Due to advancements in Helicobacter pylori treatment regimens including proton pump inhibitors (PPIs) and histamine-2 receptor antagonists (H2RAs), the incidence, hospitalization rate, and mortality associated with peptic ulcer disease have significantly declined (2).Nevertheless, 10%−20% of patients still develop PU-related complications, primarily including bleeding and perforation. Notably, perforated peptic ulcer (PPU) represents one of the most common acute abdominal conditions in surgical practice. Without early diagnosis and prompt surgical intervention, severe complications may occur, such as localized or generalized peritonitis, sepsis, and even death, with a mortality rate of approximately 9% (3). Consequently, surgery remains the most appropriate treatment for PPU. However, despite surgical management of PPU, the high incidence of postoperative complications continues to significantly impact patient prognosis, including leak/fistula formation, wound complications, and pulmonary complications. Thus, early detection and timely management of these complications, particularly severe ones, hold substantial clinical significance for postoperative recovery. Common risk factors for severe perioperative complications in PPU typically include gender, age, and patients’ general condition (particularly nutritional status, as patients with long-term gastrointestinal malignancies often present with chronic wasting conditions such as anemia and hypoproteinemia) (4, 5). Given the multitude of potential risk factors involved, employing accurate prediction tools and early intervention may represent effective strategies to improve cure rates in PPU patients. Previous studies have predominantly been single-center with limited sample sizes (6). This study aims to identify risk factors for severe postoperative complications in PPU patients and develop a predictive model based on multicenter data and an expanded cohort, thereby providing clinical evidence to guide PPU management.

## 2 Materials and methods

### 2.1 Data collection and patients’ grouping

We retrospectively analyzed medical records of 230 patients who underwent PPU repair surgery at Haiyan People's Hospital and Jiaxing Second Hospital between January 2018 and June 2024 as the training set. An independent validation set was established with 98 PPU repair cases (approximately 1:4 ratio to the training set).

Inclusion criteria: (1) Age ≥ 18years; (2) Confirmed PPU diagnosis (based on clinical manifestations, imaging findings, and intraoperative/pathological confirmation); (3) Standardized surgical treatment (laparoscopic or open approach) without complex combined procedures; (4) Postoperative hospitalization ≥48 h with completed 30-day follow-up.

Exclusion criteria: (1) Patients with severe mental disorders; (2) Surgery for non-peptic ulcer perforations or complex combined surgeries; (3) pregnancy; (4) Postoperative hospitalization <48 h, incomplete data, or lost to follow-up.

### 2.2 Factors influencing severe complication rates

Based on previous research (7) and clinical experience, we collected general clinical data and 34 potential risk variables for severe complications, including: Sex, Age, Body mass index (BMI), Smoking status, Alcohol consumption, NSAID use, Steroid use, Hypertension, Diabetes mellitus, Renal disease, Cardiovascular disease, Cancer history, Liver disease, Pathology cancer, Arrived hospital later than 24 h, Surgical approach, Operation time, Ulcer location, Ulcer diameter, ASA score, Admission nutritional score, Preoperative pain score, Enhanced recovery after surgery (ERAS), Hospital stay, Preoperative laboratory values [White blood cell count, C-reactive protein (CRP), Hemoglobin, Platelet count, Albumin, Platelet/Albumin Ratio (PAR), Creatinine, Aspartate aminotransferase (AST), Alanine aminotransferase (ALT), Procalcitonin (PCT)].

### 2.3 Definition of relevant indicators

Definition of postoperative complication severity: Assessed using the Clavien-Dindo Classification (Grade 0-V) (8). Two attending surgeons (Kui and Li) independently evaluated the classification based on patient medical records. Patients were stratified into two groups according to CDC grades: Mild complications (CDC 0-II); Severe complications (CDC III-V).

Assessment tools: Preoperative nutritional status: NRS-2002 (9), It primarily consists of three components: (1) Age (1 point if >70 years); (2) Nutritional status (BMI, reduced food intake in the past week, weight loss over 3 months); (3) Disease severity; Preoperative pain: Numerical Rating Scale (NRS); Alcohol history: Defined as >20 g alcohol consumption per week. The ASA classification system (10) stratifies patients into six categories in Table 1.

**Table 1:** The ASA classification system.

Classification	Performance
I	A normal healthy patient;
II	A patient with mild systemic disease
III	A patient with severe systemic disease that is not life-threatening
IV	A patient with severe systemic disease that is a constant threat to life
V	A moribund patient who is not expected to survive without the operation
VI	A declared brain-dead patient whose organs are being removed for donor purposes

### 2.4 Statistics and ethics

**Figure 1:**
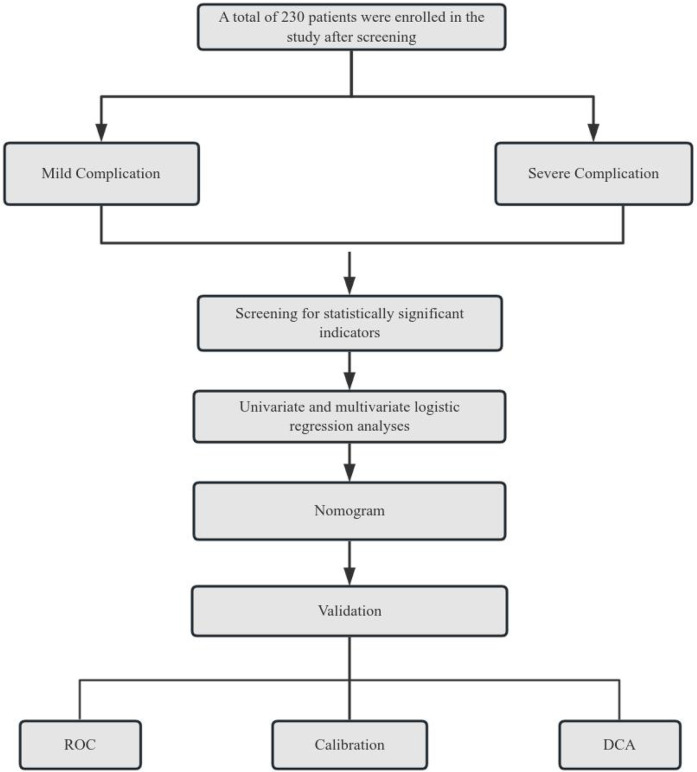
Technical roadmap.

Normally distributed continuous variables were expressed as mean ± standard deviation (x¯ ± s) and compared using independent samples t-tests. Categorical variables were described as frequencies and percentages (%) and analyzed by *χ*² test or Fisher's exact test. Ordinal data were compared using rank-sum tests. Univariate and multivariate logistic regression analyses were performed with forest plots generated to display results. A predictive model for severe complication risk was constructed using the “nomogram” function in R language. The performance of the nomogram was evaluated through both internal and external validation: The Hosmer Lemeshow test was employed to assess goodness of fit, while the area under the ROC curve (AUC) was used to validate the predictive efficacy of the model, with the Youden index determining the optimal cut-off values. Clinical utility was assessed via decision curve analysis (DCA) using the “rmda” package. All analyses were conducted using SPSS 25.0 (IBM Corp, Armonk, NY, USA) and R software (version 3.5.2). *P*-value < 0.05 was considered statistically significant. Technical Roadmap see Figure 1.

This study was approved by the Ethics Committee of Haiyan People's Hospital, Zhejiang Province (2025–08).

## 3 Results

### 3.1 Perioperative data

According to the inclusion and exclusion criteria, a total of 230 PPU patients were enrolled, comprising 185 males and 45 females with a mean age of 62.2 ± 17.8 years. The majority of patients underwent laparoscopic surgery (93.0%), including 220 cases of gastric ulcer perforation and 10 cases of duodenal ulcer perforation. Severe complications occurred in 42 cases (18.3%). Additional baseline characteristics and perioperative data are presented in Table 2.

**Table 2:** Clinical characteristics of patients.

Variable	Total (*N* = 230)	Mild complication(*N* = 188 )	Severe complication( *N* = 42 )	*p-Value*
Age (years, mean ± SD)	62.2 ± 17.8	59.0 ± 17.3	76.3 ± 12.9	<0.001
Sex				0.326
Female	45 (19.6%)	34 (18.1%)	11 (26.2%)	
Male	185 (80.4%)	154 (81.9%)	31 (73.8%)	
Body mass index (kg/m^2^)	21.4 ± 2.9	21.5 ± 2.8	21.2 ± 3.4	0.545
Smoking	92 (40.0%)	68 (36.2%)	24 (57.1%)	0.020
Alcohol	64 (27.8%)	41 (21.8%)	23 (54.8%)	<0.001
NSAID use	32 (13.9%)	19 (10.1%)	13 (31.0%)	0.001
Steroid use	6 (2.61%)	4 (2.13%)	2 (4.76%)	0.301
Hypertension	69 (30.0%)	47 (25.0%)	22 (52.4%)	0.001
Diabetes	14 (6.1%)	11 (5.9%)	3 (7.1%)	0.724
Renal disease	3 (1.3%)	1 (0.5%)	2 (4.8%)	0.087
Cardiac disease	16 (7.0%)	8 (4.3%)	8 (19.0%)	0.003
Cancer history	2 (0.9%)	1 (0.5%)	1 (2.4%)	0.333
Liver disease	6 (2.6%)	3 (1.6%)	3 (7.1%)	0.076
Pathology cancer	3 (1.3%)	2 (1.1%)	1 (2.4%)	0.496
Arrived later than 24 h	41 (17.8%)	22 (11.7%)	19 (45.2%)	<0.001
Approach method				<0.001
Open	16 (7.0%)	6 (3.2%)	10 (23.8%)	
Laparoscopic	214 (93.0%)	182 (96.8%)	32 (76.2%)	
Operation time (min, mean ± SD)	72.5 ± 39.7	65.2 ± 27.7	105 ± 63.0	<0.001
Ulcer location				0.088
Gastric	220 (95.7%)	182 (96.8%)	38 (90.5%)	
Duodenal	10 (4.4%)	6 (3.2%)	4 (9.5%)	
Ulcer diameter (cm)	1.1 ± 1.0	0.9 ± 0.7	2.0 ± 1.4	<0.001
ASA score	1.5 ± 0.8	1.3 ± 0.6	2.5 ± 1.0	<0.001
Nutrition Risk Screening	2.3 ± 0.5	2.2 ± 0.4	2.8 ± 0.5	<0.001
Pain score	3.8 ± 0.9	3.9 ± 0.8	3.3 ± 1.1	0.005
ERAS	57 (24.8%)	52 (27.7%)	5 (11.9%)	0.052
Hospital stay (mean ± SD)	11.5 ± 5.6	11.1 ± 4.3	13.2 ± 9.2	0.161
Pre WBC (×10^9^/L)	11.4 ± 5.3	11.2 ± 4.9	12.2 ± 6.9	0.416
Pre CRP (mg/L)	48.4 ± 71.5	34.4 ± 54.5	111 ± 101	<0.001
Pre HB (g/L)	124 ± 27.7	128 ± 25.1	106 ± 31.6	<0.001
Pre PLT (×10^9^/L)	240 ± 93.5	237 ± 87.5	256 ± 117	0.319
Pre Albumin (g/L)	35.8 ± 5.8	37.3 ± 4.9	29.0 ± 4.2	<0.001
Pre PAR	6.9 ± 3.1	6.4 ± 2.6	8.9 ± 4.0	<0.001
Pre Creatinine (µmol/L)	109 ± 61.6	98.3 ± 54.9	158 ± 67.3	<0.001
Pre AST (U/L)	26.0 ± 30.5	22.8 ± 18.7	40.5 ± 57.8	0.057
Pre ALT (U/L)	26.9 ± 21.3	24.8 ± 18.7	36.5 ± 28.8	0.015
Pre PCT (ng/ml)	7.39 (2.84, 13.1)	7.10 (2.75, 11.2)	10.1 (3.21, 21.6)	0.033

ERAS, enhanced recovery after surgery.

### 3.2 Risk factors for severe postoperative complications

Univariate logistic regression identified 23 risk factors associated with severe postoperative complications after PPU repair, including: age, smoking history, alcohol, NSAID use, hypertension, cardiovascular disease, Arrived hospital later than 24 h, surgical approach, operation time, ulcer diameter, ASA score, nutritional score, preoperative pain score, ERAS, hospital stay, and preoperative laboratory markers (CRP, hemoglobin, albumin, PAR, creatinine, AST, ALT, PCT). Variables with statistical significance (*P* < 0.05) in univariate analysis were included in multivariate logistic regression, which revealed alcohol, ASA score, nutritional score, and preoperative albumin level as independent risk factors for severe complications (*P* < 0.05, in Table 3).

**Table 3:** Factors that associated with severe complications.

Variable	Univariable			Multivariable		
	Odds Ratio	95% CI	*p-Value (<0.05)*	Odds Ratio	95% CI	*p-Value (<0.05)*
Age	1.08	(1.05–1.11)	<0.001	1.03	(0.96–1.11)	0.376
Sex	0.62	(0.28–1.36)	0.234			
BMI	0.96	(0.85–1.08)	0.495			
Smoking	2.35	(1.19–4.64)	0.014	0.52	(0.08–3.38)	0.49
Alcohol	4.34	(2.16–8.73)	<0.001	10.18	(1.50–69.16)	0.018
NSAID use	3.99	(1.78–8.95)	<0.001	2.64	(0.31–22.14)	0.371
Steroid use	2.3	(0.41–12.99)	0.346			
Hypertension	3.3	(1.66–6.58)	<0.001	0.69	(0.12–3.85)	0.673
Diabetes	1.24	(0.33–4.65)	0.752			
Renal disease	9.35	(0.83–105.64)	0.071			
Cardiac disease	5.29	(1.86–15.07)	0.002	0.66	(0.07–6.48)	0.72
Cancer history	4.56	(0.28–74.43)	0.287			
Liver disease	4.74	(0.92–24.38)	0.062			
Arrived later than 24 h	0.16	(0.08–0.34)	<0.001	0.44	(0.07–2.87)	0.388
Approach method	0.11	(0.04–0.31)	<0.001	0.6	(0.04–10.04)	0.722
Operation time	1.02	(1.01–1.04)	<0.001	1.01	(0.99–1.03)	0.475
Ulcer location	3.19	(0.86–11.86)	0.083			
Ulcer diameter	2.74	(1.86–4.06)	<0.001	0.93	(0.45–1.93)	0.849
ASA score	4.78	(3.06–7.47)	<0.001	1.58	(0.32–7.92)	0.576
Nutrition Risk Screening	11.58	(5.18–25.85)	<0.001	4.27	(0.46–39.52)	0.201
Pain score	0.49	(0.33–0.73)	<0.001	1.77	(0.72–4.35)	0.212
ERAS	0.35	(0.13–0.95)	0.039	0.67	(0.10–4.65)	0.685
Hospital stay	1.05	(1.00–1.11)	0.042	0.99	(0.88–1.12)	0.876
Pre WBC	1.03	(0.97–1.10)	0.308			
Pre CRP	1.01	(1.01–1.02)	<0.001	1.01	(1.00–1.02)	0.005
Pre HB	0.97	(0.96–0.98)	<0.001	0.99	(0.96–1.02)	0.451
Pre PLT	1	(1.00–1.01)	0.229			
Pre Albumin	0.7	(0.63–0.78)	<0.001	0.75	(0.63–0.91)	0.003
Pre PAR	1.28	(1.14–1.42)	<0.001	1.05	(0.85–1.29)	0.677
Pre Creatinine	1.01	(1.01–1.02)	<0.001	1	(0.99–1.02)	0.614
Pre AST	1.01	(1.00–1.03)	0.008	0.98	(0.94–1.02)	0.386
Pre ALT	1.02	(1.01–1.03)	0.003	1.03	(0.98–1.09)	0.218
Pre PCT	1.02	(1.01–1.03)	<0.001	1.01	(0.98–1.04)	0.517

### 3.3 Development and validation of the nomogram

**Figure 2:**
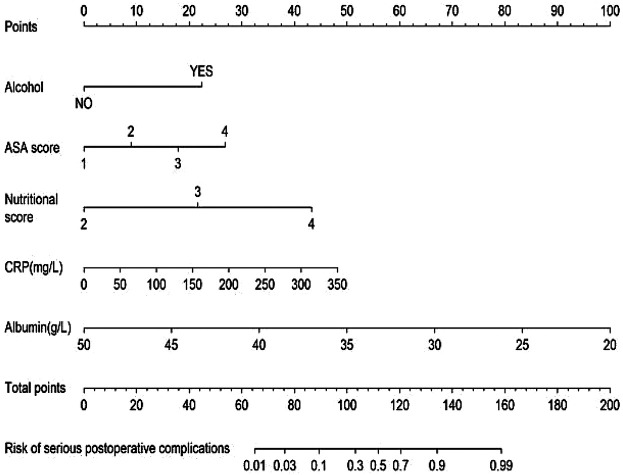
Nomogram for severe complications.

**Figure 3:**
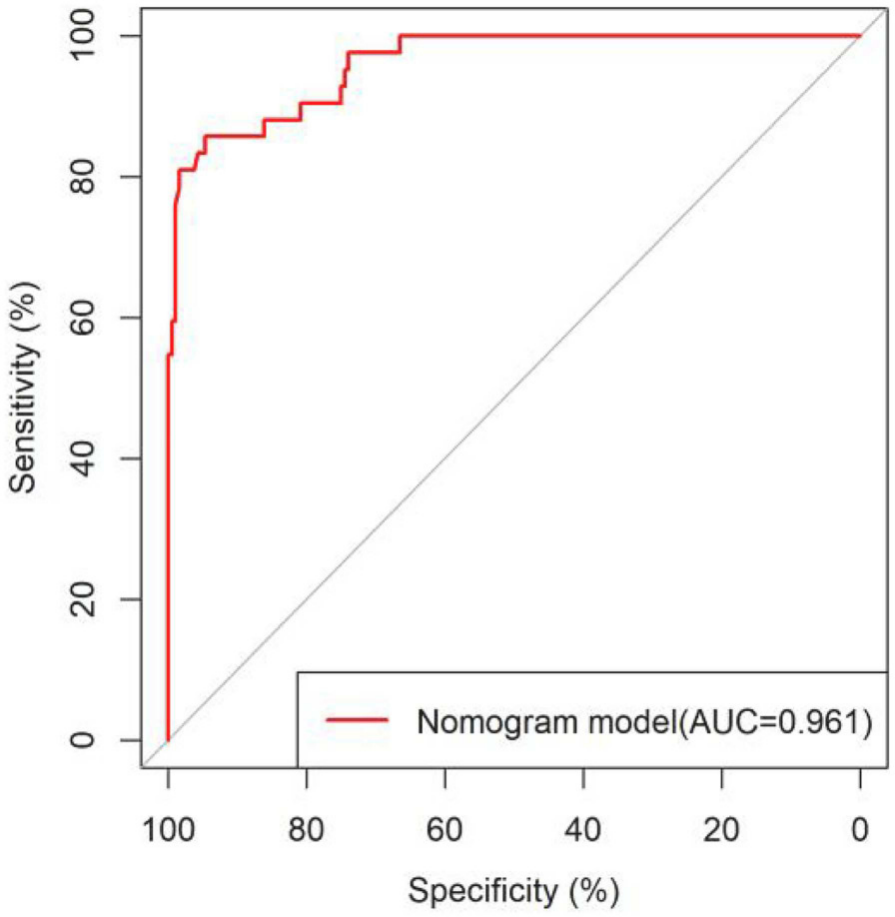
ROC curve of the internal dataset.

**Figure 4:**
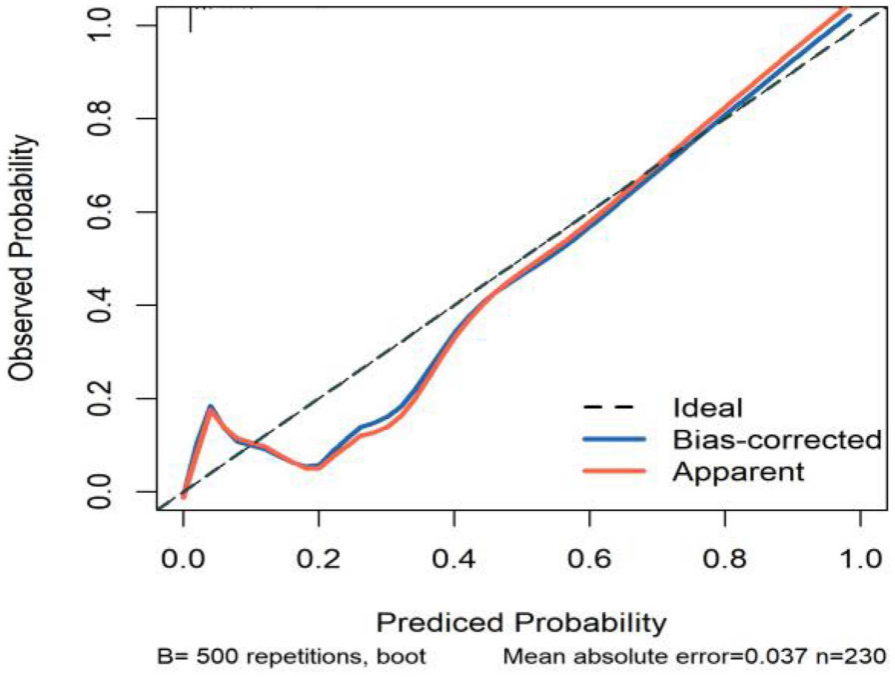
Calibration curve of the internal dataset.

**Figure 5:**
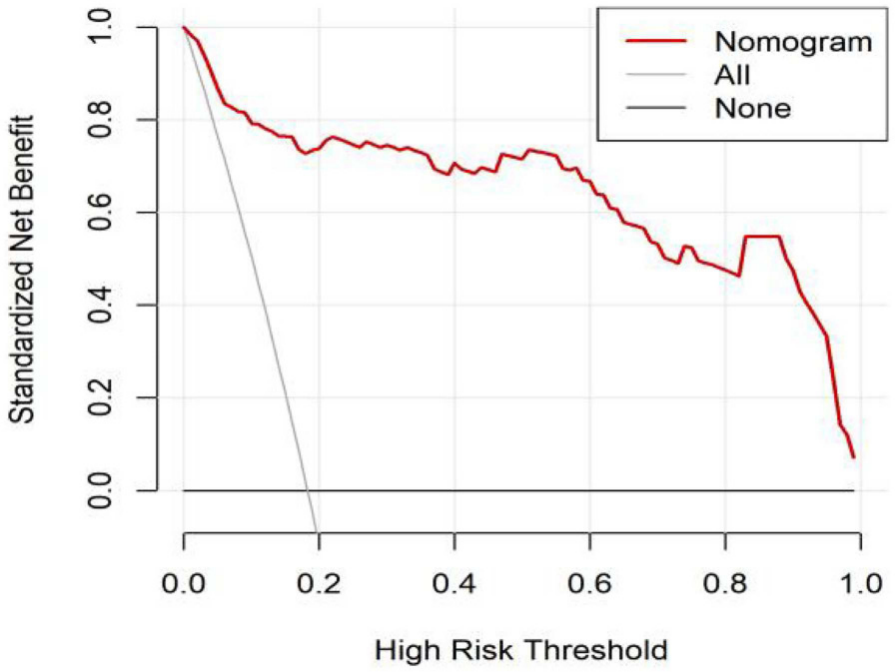
DCA curve of the internal dataset.

All significant risk factors identified in the multivariate analysis were incorporated to construct a predictive model for severe complication risk, with a corresponding nomogram developed (Figure 2). Internal validation results showed that the model had good goodness of fit (*P* = 0.3451), excellent calibration, and good discriminative ability (AUC was 0.961, 95% CI: 0.769–0.894, Sensitivity: 82.36%, Specificity:73.86%). These results confirm the model's robust reliability and clinical validity (Figure 3). The calibration plot revealed good agreement between predicted and observed outcomes, with a mean absolute error (MAE) of 0.037 between predicted and actual probabilities, indicating optimal calibration performance (Figure 4). Decision curve analysis (DCA) was performed with net benefit rate as the *y*-axis and high risk threshold probability as the *x*-axis. The results showed positive net benefit rates (net benefit >0) across the entire probability range (0–1.0), confirming the model's clinical utility (Figure 5).

**Figure 6:**
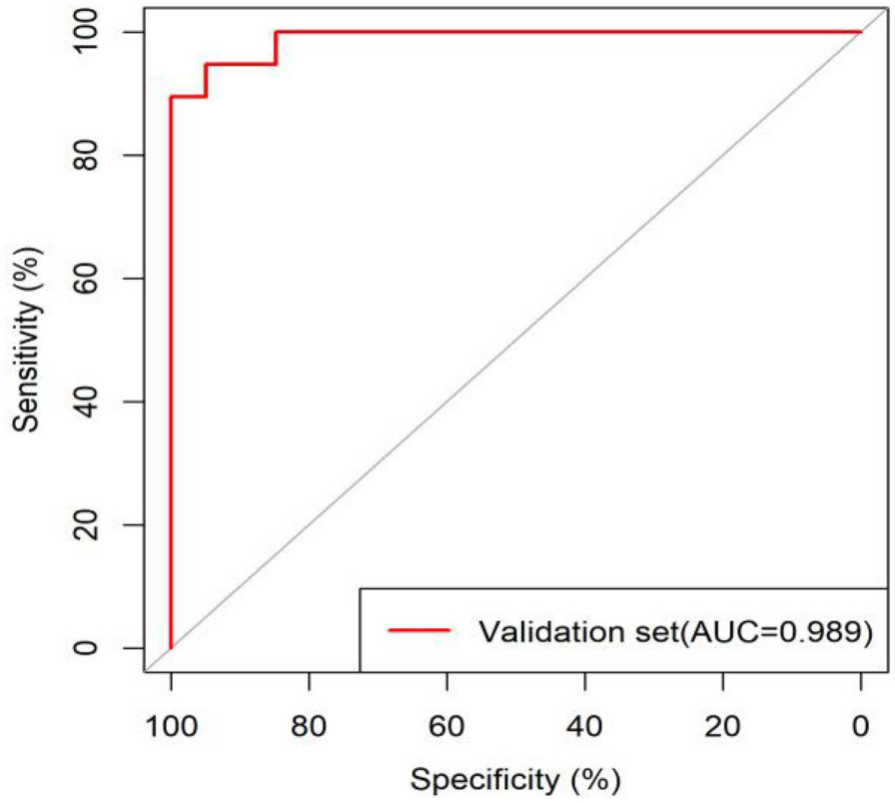
ROC curve of the external dataset.

**Figure 7:**
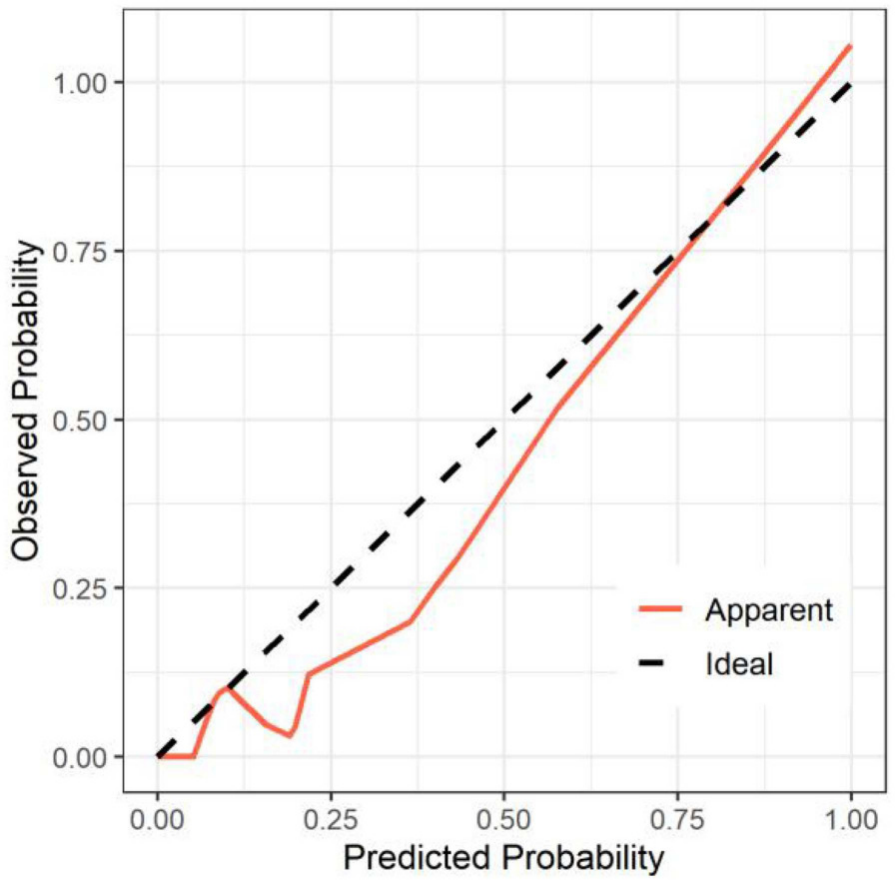
Calibration curve of the external dataset.

**Figure 8:**
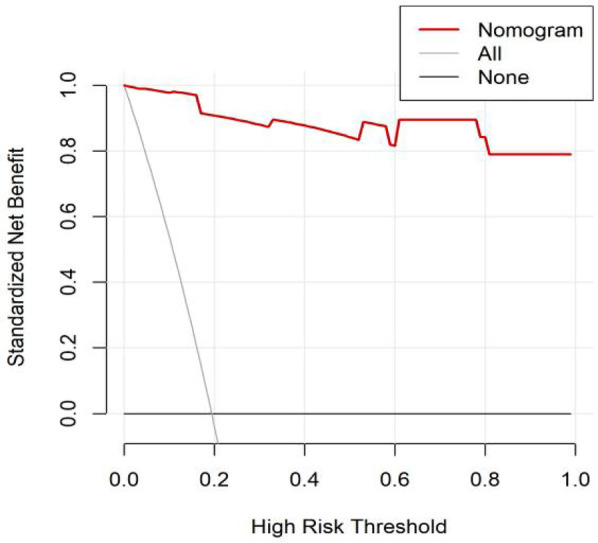
DCA curve of the external dataset.

External validation results showed that the model had good goodness of fit (*P* = 0.9913), excellent calibration, and good discriminative ability (AUC was 0.989, 95% CI: 0.972–1.000, Sensitivity:94.7%, Specificity: 94.9%). Calibration curve analysis revealed strong agreement between predicted and observed probabilities, indicating superior model calibration. Decision curve analysis (DCA) demonstrated positive net benefit (net benefit rate >0) across the entire probability threshold range (0–1.0), indicating robust clinical utility of the model (Figures 6–8).

## 4 Discussion

Peptic ulcer (PU) represents one of the most prevalent gastrointestinal disorders. As a severe complication of PU, perforated peptic ulcer (PPU) most frequently occurs in individuals aged 40–50 years. PPU can rapidly progress to acute abdomen, leading to life-threatening complications including peritonitis, sepsis, and mortality (11). Although more prevalent in males, the male-to-female ratio shows significant geographical variation. Both mortality and morbidity rates demonstrate an age-dependent increase, with particularly steep escalation beyond 60 years of age (12, 13). Severe postoperative complications occurred in approximately 18.3% of patients in our cohort. Previous studies have reported a complication rate ranging from 9.1%–17% following PPU repair surgery (14, 15).

The occurrence of severe complications significantly impacts patient prognosis, making early detection and prevention clinically crucial. However, accurate early prediction of severe complications remains challenging. This study aimed to establish a reliable predictive model to identify risk factors for severe complications, thereby providing clinicians with evidence-based tools for early detection. Timely intervention based on these predictions may mitigate the negative impact of severe complications on patient outcomes and ultimately improve quality of life.

A 2016 study by Sivaram et al. demonstrated that Female gender, older age group, perforation surgery interval more than 36 h, and size of perforation more than 1 cm^2^ were found to be significant factors influencing postoperative mortality and morbidity. Postoperative morbidity was also associated with comorbid diseases. Abnormal renal function on presentation was identified as an additional risk factor for postoperative morbidity and longer hospital stay (6).

### 4.1 Alcohol

The causal relationship between alcohol consumption and peptic ulcer (PU) development remains controversial. Nolan J and Jenkins RA et al. (16) reported that chronic alcohol users exhibit higher PU incidence than the general population. Heavy alcohol intake causes significant gastric epithelial damage, deep mucosal necrosis, and microvascular injury leading to congestion, increased permeability, and intramucosal hemorrhage. Beyond local irritant effects, ethanol at moderate-to-high doses has been shown to delay gastric emptying, though large-scale studies are lacking. Conversely, Shimamoto et al. found no association between alcoholic beverage consumption and PU incidence, except in patients with elevated Helicobacter pylori IgG antibodies where alcohol may potentiate risk (17). Our study identifies prior alcohol use as an independent risk factor significantly increasing severe postoperative complications.

### 4.2 ASA score

Since its establishment in 1962 by the American Society of Anesthesiologists (ASA), the ASA Physical Status Classification System has been widely adopted in clinical practice to assess patients’ overall health status prior to undergoing surgery or anesthesia (18). Substantial evidence confirms a strong correlation between ASA classification and postoperative recovery capacity.

Patients with higher ASA classifications demonstrate: 1. Impaired early mobilization due to diminished physiological reserve; 2. Multifaceted postoperative challenges including: Suboptimal pain control, Compromised activity tolerance, Limited capacity for self-care; 3. Increased requirements for rehabilitation duration and nursing care intensity. Notably, patients with ASA class ≥ III exhibit: Preoperative functional impairment (particularly cardiopulmonary reserve); Significantly elevated risks of postoperative complications (19) including: Surgical site infections, Respiratory insufficiency, Cardiac events, Other life-threatening conditions. The research by Nertila et al. (18) revealed the association between ASA classification and postoperative recovery, providing substantial evidence for clinical decision-making. Our study further confirms the negative impact of ASA scores on patient prognosis—higher ASA scores correlate with increased probability of severe complications after PPU surgery, serving as an independent risk factor. These findings emphasize the importance of preoperative ASA evaluation and guide medical staff in developing individualized postoperative rehabilitation plans according to different ASA classifications. This approach can effectively reduce postoperative complications, improve patients’ quality of life, and optimize the use of medical resources.

### 4.3 Preoperative nutritional score and albumin level

The Nutritional Risk Screening 2002 (NRS-2002) was developed by ESPEN in June 2002 based on multiple RCT evidence. The total score ranges from 0–7 points. An NRS-2002 score ≥3 indicates nutritional risk and necessitates nutritional support intervention (9). Patients frequently exhibit varying degrees of metabolic disturbances and malnutrition across different treatment phases (20, 21). Malnutrition will affect patients’ surgical decision-making and treatment outcomes, increase postoperative complication rates, slow down recovery speed, reduce quality of life, and lead to multiple adverse clinical outcomes for patients.

Serum albumin level is a key indicator for assessing patients’ nutritional status and healing capacity. Hypoalbuminemia compromises patients’ immunity and increases surgical risks. Tu and Lin et al. suggest the underlying mechanism may be that surgical trauma leads to tissue edema and increased fragility, resulting in impaired granulation tissue formation, reduced healing capacity, and elevated infection risk (22). The findings of this study demonstrate that serum albumin level is an independent risk factor for severe postoperative complications. This result is consistent with the findings of Yun-Suk Choi et al. (7).

Therefore, correcting hypoalbuminemia is particularly important in perioperative management. Furthermore, current research recommends using indicators such as prealbumin and transferrin to more accurately assess patients’ nutritional status (12), as these markers can more sensitively reflect changes in protein metabolism. Prealbumin has a shorter half-life (2 days) compared to albumin (20–25 days) and shows more significant decreases under surgical stress and negative nitrogen balance, making it useful for monitoring nutritional depletion. Thus, in clinical practice, in addition to monitoring traditional albumin levels, clinicians should also consider incorporating other nutritional markers like prealbumin to comprehensively evaluate patients’ nutritional status and healing potential.

Furthermore, it is important to note that other validated nutritional screening tools exist, some of which may offer higher sensitivity and specificity. For instance, the Malnutrition Universal Screening Tool (MUST) was identified in a large multicentre prospective study as the most valid tool for both diagnosing malnutrition and predicting postoperative outcomes in surgical patients (23). This approach would enhance the scientific rigor and clinical applicability of research findings, while providing evidence-based support for selecting the most appropriate assessment tools for different patient populations.

Regarding nutritional management, another important approach is Enhanced Recovery After Surgery (ERAS), which optimizes perioperative care based on the characteristics of metabolic changes, aiming to reduce surgical trauma and stress response, and promote rapid recovery (24). Perioperative nutritional therapy under the ERAS protocol should regulate patients’ metabolism to mitigate surgical stress-induced damage and gastrointestinal dysfunction, enhance protein synthesis, reduce skeletal muscle catabolism, and help the body adapt to metabolic stress changes. This approach prevents and treats catabolism and malnutrition, maintains surgical patients’ perioperative nutritional status, and reduces postoperative complications.

Our study performed statistical analysis on the effects of ERAS protocols during the perioperative period of PPU patients. The results demonstrated that the ERAS group showed advantages in the following aspects: postoperative pain, Sipping water start time, Activity start time, and BMI at discharge (Table 4). Although no significant differences were observed in length of hospital stay or incidence of severe complications, existing robust evidence supports the positive effects of ERAS in disease treatment and recovery (25). Therefore, further research and discussion are needed to fully evaluate the advantages and limitations of ERAS for PPU patients.

**Table 4:** Stratified analysis based on ERAS protocol implementation status.

Variable	NO ERAS(*N* = 164)	ERAS( *N* = 62)	*p-Value*
Postoperative pain score	2.3 ± 0.51	2.13 ± 0.46	0.018
Sipping water start time (postoperative days)	7.4 ± 1.01	6.79 ± 1.15	<0.001
Activity start time(postoperative days)	2.19 ± 0.87	1.27 ± 0.61	<0.001
Hospital stay (day)	11.66 ± 5.94	11.56 ± 4.03	0.91
Discharge nutritional score	2.79 ± 1.64	3.35 ± 1.70	0.022
Discharge BMI	20.20 ± 2.65	21.10 ± 3.27	0.033
Severe complication	28 (17.1%)	10 (16.1%)	0.866

### 4.4 Preoperative CRP level

C-reactive protein (CRP) is an acute-phase reactive protein that can form complexes with pneumococcal C-polysaccharide. It belongs to a group of plasma proteins that rapidly increase during infection or tissue injury. CRP plays a crucial protective role in innate immunity by activating complement and enhancing phagocytosis, thereby eliminating invading pathogens as well as damaged, necrotic, and apoptotic cells. Elevated preoperative CRP levels in perforated peptic ulcer patients may increase the likelihood of postoperative complications by impairing tissue perfusion. Common pulmonary infections can elevate inflammatory markers, potentially leading to persistent hypoxemia or even respiratory failure. Several studies have demonstrated the utility of serum CRP levels in predicting complication severity before clinical signs or symptoms appear (6, 7). This study yielded similar findings, showing that higher preoperative CRP levels correlate with increased incidence of severe postoperative complications.

## 5 Conclusion

The findings of this study demonstrate that alcohol use history, ASA score, admission nutritional score, CRP level, and preoperative albumin level were independent risk factors for severe complications. The model demonstrated good performance in both internal and external validation with the current data but has limitations including relatively small sample size and lack of large-scale external validation data from other institutions. Therefore, the model's generalizability requires further evaluation. Future studies should focus on multicenter, large-sample, and well-designed research to further validate the reliability of the nomogram prediction model.

## Data Availability

The original contributions presented in the study are included in the article/Supplementary Material, further inquiries can be directed to the corresponding author.

## References

[1] Lanas A Chan FKL. Peptic ulcer disease. Lancet. (2017) 390(10094):613–24. 10.1016/S0140-6736(16)32404-7 28242110

[2] Almadi MA Lu Y Alali AA Barkun AN. Peptic ulcer disease. Lancet. (2024) 404(10447):68–81. 10.1016/S0140-6736(24)00155-7 38885678

[3] Abouelazayem M Jain R Wilson MSJ Martinino A Balasubaramaniam V Biffl W Global 30-day morbidity and mortality of surgery for perforated peptic ulcer: GRACE study. Surg Endosc. (2024) 38(8):4402–14. 10.1007/s00464-024-10881-0 38886232

[4] Celik SU Gulap Y Demir MB Demircioglu MM Polat HE Kesikli SA. Predictive value of the red cell distribution width-to-albumin ratio for clinical outcomes in patients with peptic ulcer perforation. World J Surg. (2025) 49(4):873–80. 10.1002/wjs.12515 39993970 PMC11994152

[5] Shreya A Sahla S Gurushankari B Shivakumar M Rifai Kate V Spectrum of perforated peptic ulcer disease in a tertiary care hospital in South India: predictors of morbidity and mortality. ANZ J Surg. (2024) 94(3):366–70. 10.1111/ans.18831 38115644

[6] Sivaram P Sreekumar A. Preoperative factors influencing mortality and morbidity in peptic ulcer perforation. Eur J Trauma Emerg Surg. (2018) 44(2):251–7. 10.1007/s00068-017-0777-7 28258286

[7] Choi YS Heo YS Yi JW. Clinical characteristics of primary repair for perforated peptic ulcer: 10-year experience in a single center. J Clin Med. (2021) 10(8):1790. 10.3390/jcm10081790 33924059 PMC8073572

[8] Clavien PA Barkun J de Oliveira ML Vauthey JN Dindo D Schulick RD The Clavien-Dindo classification of surgical complications: five-year experience. Ann Surg. (2009) 250(2):187–96. 10.1097/SLA.0b013e3181b13ca2 19638912

[9] Kondrup J Rasmussen HH Hamberg OLE Stanga Z. Nutritional risk screening (NRS 2002): a new method based on an analysis of controlled clinical trials. Clin Nutr. (2003) 22(3):321–36. 10.1016/S0261-5614(02)00214-5 12765673

[10] Dripps RD Lamont A Eckenhoff JE. The role of anesthesia in surgical mortality. JAMA. (1961) 178:261–6. 10.1001/jama.1961.03040420001001 13887881

[11] Søreide K Thorsen K Harrison EM Bingener J Møller MH Ohene-Yeboah M Perforated Peptic Ulcer. Lancet. (2015) 386(10000):1288–98. 26460663 10.1016/S0140-6736(15)00276-7 PMC4618390

[12] Zittel TT Jehle EC Becker HD. Surgical management of peptic ulcer disease today–indication, technique and outcome. Langenbecks Arch Surg. (2000) 385(2):84–96. 10.1007/s004230050250 10796046

[13] Kocer B Surmeli S Solak C Unal B Bozkurt B Yildirim O Factors affecting mortality and morbidity in patients with peptic ulcer perforation. J Gastroenterol Hepatol. (2007) 22(4):565–70. 10.1111/j.1440-1746.2006.04500.x 17376052

[14] Quah GS Eslick GD Cox MR. Laparoscopic repair for perforated peptic ulcer disease has better outcomes than open repair. J Gastrointest Surg. (2019) 23(3):618–25. 10.1007/s11605-018-4047-8 30465190

[15] Wilhelmsen M Møller MH Rosenstock S. Surgical complications after open and laparoscopic surgery for perforated peptic ulcer in a nationwide cohort. Br J Surg. (2015) 102(4):382–7. 10.1002/bjs.9753 25605566

[16] Andersen IB Jørgensen T Bonnevie O Grønbaek M Sørensen TI. Smoking and alcohol intake as risk factors for bleeding and perforated peptic ulcers: a population-based cohort study. Epidemiology (Cambridge, Mass). (2000) 11(4):434–9. 10.1097/00001648-200007000-00012 10874551

[17] Shimamoto T Yamamichi N Kodashima S Takahashi Y Fujishiro M Oka M No association of coffee consumption with gastric ulcer, duodenal ulcer, reflux esophagitis, and non-erosive reflux disease: a cross-sectional study of 8,013 healthy subjects in Japan. PLoS One. (2013) 8(6):e65996. 10.1371/journal.pone.0065996 23776588 PMC3680393

[18] Kodra N Shpata V Ohri I. Risk factors for postoperative pulmonary complications after abdominal surgery. Open Access Maced J Med Sci. (2016) 4(2):259–63. 10.3889/oamjms.2016.059 27335597 PMC4908742

[19] Watanabe M Kinoshita T Tokunaga M Kaito A Sugita S. Complications and their correlation with prognosis in patients undergoing total gastrectomy with splenectomy for treatment of proximal advanced gastric cancer. Eur J Surg Oncol. (2018) 44(8):1181–5. 10.1016/j.ejso.2018.03.013 29610022

[20] Lobo DN Gianotti L Adiamah A Barazzoni R Deutz NEP Dhatariya K Perioperative nutrition: recommendations from the ESPEN expert group. Clin Nutr. (2020) 39: 3211–27. 10.1016/j.clnu.2020.03.038 32362485

[21] Ford KL Prado CM Weimann A Schuetz P Lobo DN. Unresolved issues in perioperative nutrition: a narrative review. Clin Nutr. (2022) 41(7):1578–90. 10.1016/j.clnu.2022.05.015 35667274

[22] Tu RH Lin JX Zheng CH Li P Xie JW Wang JB Development of a nomogram for predicting the risk of anastomotic leakage after a gastrectomy for gastric cancer. Eur J Surg Oncol. (2017) 43(2):485–92. 10.1016/j.ejso.2016.11.022 28041649

[23] Petra G Kritsotakis EI Gouvas N Schizas D Toutouzas K Karanikas M Multicentre prospective study on the diagnostic and prognostic validity of malnutrition assessment tools in surgery. Br J Surg. (2025) 112(2):znaf013. 10.1093/bjs/znaf013 40037524 PMC11879291

[24] Sun YM Wang Y Mao YX Wang W. The safety and feasibility of enhanced recovery after surgery in patients undergoing pancreaticoduodenectomy: an updated meta-analysis. BioMed Res Int. (2020) 2020:7401276. 10.1155/2020/7401276 32462014 PMC7232716

[25] Zeyara A Thomasson J Andersson B Tingstedt B. Fast-track recovery after surgery for perforated peptic ulcer safely shortens hospital stay: a systematic review and meta-analysis of six randomized controlled trials and 356 patients. World J Surg. (2024) 48(7):1575–85. 10.1002/wjs.12234 38838070

